# The impact of 3 different dietary interventions on overweight or obese adults: A network meta-analysis

**DOI:** 10.1097/MD.0000000000039749

**Published:** 2024-10-18

**Authors:** Tianrong Liao, Jiayu Su, Tingwei Quan, Yu Luo, Yiqian Zeng, Dandan Chen, Hongzhen Tang

**Affiliations:** a Department of Acupuncture and Tuina, Guangxi University of Chinese Medicine, Nanning, Guangxi Province, China; b Department of Acupuncture and Tuina, Zhaoqing Hospital of Chinese Medicine Affiliated to Southern Medical University, Zhaoqing, Guangdong Province, China; c Department of Laser Plastic Surgery, The First Affiliated Hospital of Guangxi University of Chinese Medicine, Nanning, Guangxi Province, China; d Department of Acupuncture and Tuina, Ruikang Hospital Affiliated to Guangxi University of Chinese Medicine, Nanning, Guangxi Province, China.

**Keywords:** adults, dietary interventions, network meta-analysis, obesity/overweight, randomized controlled trial

## Abstract

**Objective:**

This network meta-analysis aims to investigate and compare the effectiveness of 3 dietary interventions – Mediterranean, ketogenic, and low-fat diet – on overweight and obese adults, with a comparison to traditional low-calorie diet.

**Methods:**

A systematic review was conducted in both Chinese and English databases, including the China National Knowledge Infrastructure (CNKI), Wanfang Database, China Science and Technology Journal Database (VIP), SinoMed, PubMed, Web of Science, Cochrane Library and Embase to identify relevant randomized controlled trials (RCTs) up to January 31, 2024. Two researchers independently screened and extracted data from the identified literature. The quality of these studies was assessed using the Cochrane bias risk assessment tool. A random-effects network meta-analysis was performed using Review Manager 5.4.1 and Stata 16.0 software.

**Results:**

A total of 17 randomized controlled trials involving 5802 subjects were included in this study. The network meta-analysis revealed a descending order of effectiveness for reducing body weight (BW), body mass index (BMI), and waist circumference (WC): ketogenic diet > low-fat diet > low-calorie diet > Mediterranean diet.

**Conclusions:**

The ketogenic diet was identified as the most effective intervention for reducing BW, BMI, and WC in the studied dietary comparisons. It consistently showed superior outcomes, ranking highest in effectiveness among the 4 evaluated dietary approaches. Nevertheless, additional high-quality randomized controlled trials are necessary to validate these findings.

## 1. Introduction

Obesity is a complex health issue characterized by excessive body fat accumulation, influenced by genetic, environmental, and behavioral factors. The latest statistics from the World Obesity Alliance’s “2024 World Obesity Map” indicate that the global count of overweight and obese adults has exceeded 2.2 billion, with a continually rising obesity rate projected to reach 3.3 billion by 2035, accounting for over 54% of the global adult population.^[1]^ Obesity escalates the risk of numerous chronic diseases, including type 2 diabetes, hypertension cardiovascular diseases, kidney and liver diseases, and certain cancers.^[2]^ Moreover, the rising prevalence of obesity impairs overall quality of life, potentially leading to psychological issues and social repercussions.^[3]^

Dietary adjustment is a crucial approach to obesity management. Recent decades have seen a shift in nutritional research from focusing only on single nutrients and foods to examining the combined effects of dietary patterns, acknowledging the potential synergistic and/or antagonistic interactions within food consumption patterns.^[4,5]^ Consequently, various dietary interventions like ketogenic,^[6–8]^ low-fat,^[9]^ and Mediterranean diets^[10–12]^ have been explored for their effectiveness on weight loss. However, a direct comparative analysis of these intervention remains scant. Through network meta-analysis, this study quantitatively assesses the effectiveness of different dietary interventions, facilitating the determination of the most effective dietary pattern for obesity management.

## 2. Methods

The study adhered to the Preferred Reporting Items for Systematic Reviews and Meta-Analyses (PRISMA) guidelines^[13]^ and was registered with PROSPERO (registration ID: CRD42024514501).

### 2.1. Search strategy

The search strategy employed Medical Subject Headings (MeSH), title/abstract keywords, and free text search terms to compile data from databases including the China National Knowledge Infrastructure (CNKI), Wanfang Database, China Science and Technology Journal Database (VIP), SinoMed, PubMed, Web of Science, Cochrane Library and Embase, covering the period from their establishment to January 31, 2024. Search terms included: “adult,” or “adults”; “obesity,” “overweight,” “over fat,” “body weight,” “weight loss,” or “weight reduction”; “Mediterranean diet,” “Ketogenic diet,” or “Low-fat diet”; “randomized controlled trial,” “Randomized,” “Placebo,” or “RCT.” References of included studies were also reviewed to identify additional relevant research. Details of the search strategy for each database are provided in Supplemental File 1, Supplemental Digital Content. http://links.lww.com/MD/N594

### 2.2. Eligibility criteria

The inclusion and exclusion criteria were established using the PICOS framework (population, intervention, comparison, outcome, study design) as recommended by the Cochrane review guidelines.^[14]^ The studies were selected based on the following characteristics: Population (P): adults aged over 18 years with overweight or obesity; Intervention (I): Mediterranean diet, ketogenic diet, low-fat diet; Comparison (C): any control group in randomized control trials (RCTs) employing the Mediterranean diet, ketogenic diet, low-fat diet, or other diets; Outcome (O): BW, BMI, and WC; and Study Design (S): RCTs.

Exclusion criteria were: studies without the specified diets as an intervention or control; studies including participants with metabolic syndrome, prediabetes, type 2 diabetes or who have undergone bariatric surgery; studies with incomplete data or significant errors; and Studies where the full text was inaccessible or data could not be extracted.

### 2.3. Study selection and screening

Two authors (TW and JY) independently screened the articles based on the eligibility criteria. Duplicate articles were removed, and the remaining articles were evaluated by title and abstract for relevance. Subsequently, full-text assessments were conducted for studies deemed potentially eligible. Any disagreements between the authors (TW, JY, and LY) were resolved through discussion among 3 reviewers to reach a consensus.

### 2.4. Data extraction

Two researchers independently screened the search results and assessed the eligibility of the full-text reports. Disagreement was resolved by consensus or intervention of a third researcher.

Extracted data included: the author’s name and year of publication; country of intervention; characteristics of subjects, including the average age, baseline BMI, and sample size of the intervention and control group; type of intervention; duration; outcome indicators, including BW, BMI and WC. Efforts were made to contact original study authors for missing data.

### 2.5. Quality assessment

Two reviewers independently assessed the risk of bias in the included RCTs using the bias risk tool of the Cochrane Collaboration,^[15]^ which convers aspects such as random sequence generation, allocation concealment, blinding of subjects and personnel, blinding of outcome assessment, incomplete outcome data, selective outcome reporting, and other potential biases. Studies were categorized as having low, unclear, and high risk of bias, with any discrepancies resolved through discussion until consensus was achieved.

### 2.6. Data synthesis and statistical analysis

An exploratory random-effects network meta-analysis was conducted to allow indirect comparisons among the intervention.^[16]^ The mean difference (MD) was the measure of effect for BW, BMI, and WC. Combined effects were presented as effect values with 95% confidence intervals (95% CI). Review Manager 5.4.1 software was used for quality assessment of the included studies, and Stata 16.0 for network and inconsistency analysis, including node-splitting method to assess discrepancies between direct and indirect comparisons.^[17,18]^ The statistical analysis was based on a frequency framework, utilizing Stata 16.0 software and its network package to generate network and funnel diagram. Surface under the cumulative ranking curve (SUCRA)^[19]^ was calculated to determine the relative effectiveness of each diet, and ranking diagram were created to identify the most effective dietary intervention.

## 3. Results

### 3.1. Literature search results

**Figure 1.**
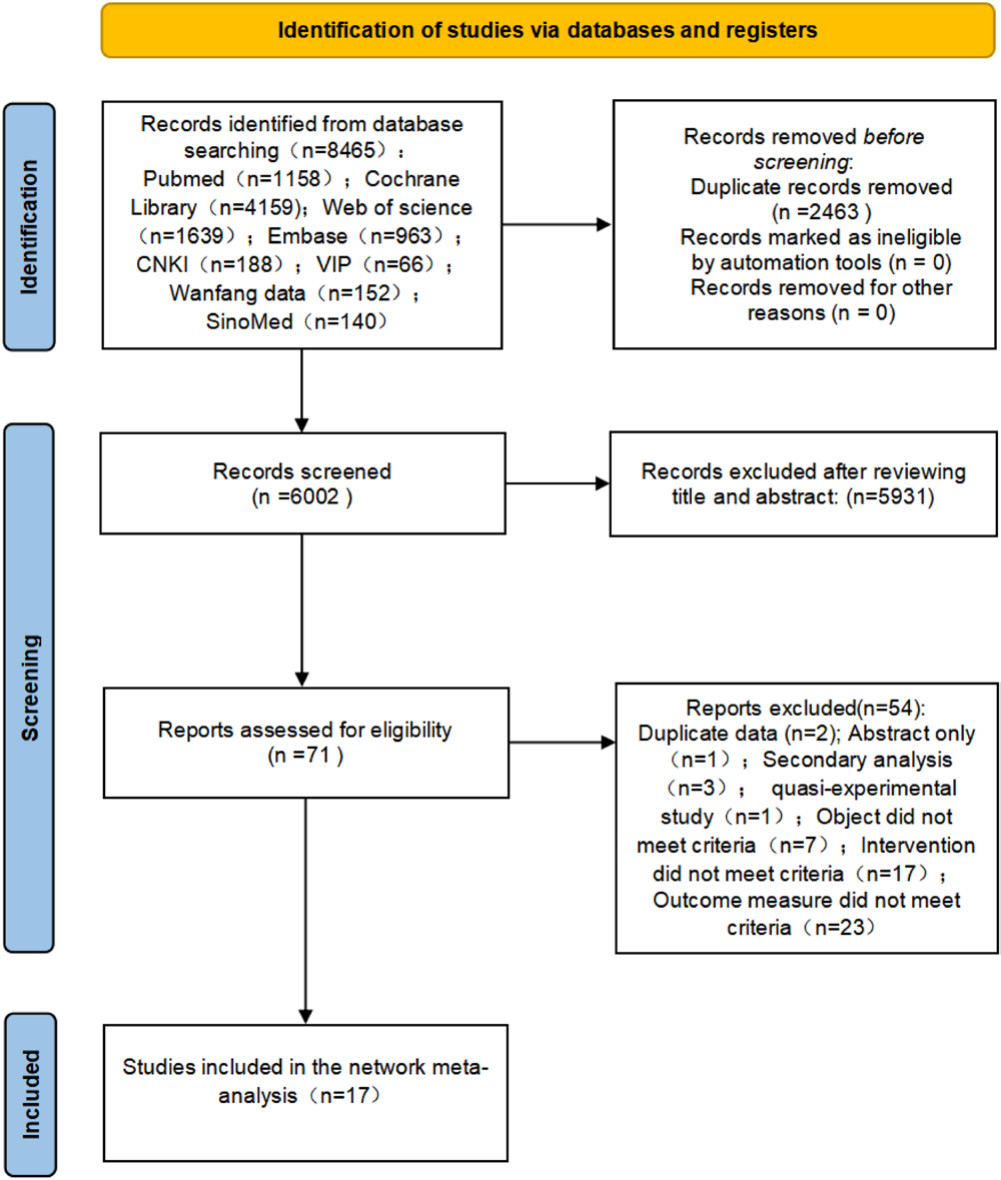
PRISMA flowchart of the study selection process. CNKI = China National Knowledge Infrastructure, PRISMA = Preferred Reporting Items for Systematic Reviews and Meta-Analyses, VIP = Wanfang Database, China Science and Technology Journal Database.

The search yielded 8465 articles, of which 6002 remained after duplicates were removed. An initial screening of titles and abstracts narrowed the selection to 71 potentially relevant articles. Upon detailed evaluation against the inclusion and exclusion criteria, 17 articles were ultimately included in the study (Fig. 1).

### 3.2. Characteristics of included studies

The characteristics of the 17 included studies are summarized in Table 1. These studies were geographically diverse, with 3 conducted in Greece,^[20–22]^ 8 in the United States,^[24,27–29,33–36]^ 2 in Australia,^[30,32]^ and 1 each in Rome,^[23]^ Spain,^[25]^ Poland,^[26]^ and across multiple European countries.^[31]^

**Table 1:** Baseline characteristics of the included studies.

Study	Country	Age, mean ± SD (I/C)	BMI (kg/m^2^), mean ± SD (I/C)	Sample size (I/C)	Intervention (type)	Duration (wk)	Outcome indicators
Dellis et al. ^[20]^	Athens, Greece	51.20 ± 10.42/48.71 ± 10.97	33.93 ± 3.66/33.40 ± 4.15	35/35	†/*	8	# ** ††
Hassapidou et al^[21]^	Greece	54.3 ± 16.4/53.9 ± 13.4	29.9 ± 5.4/32.3 ± 5.9	2210/1816	†/*	24	# ** ††
Feidantsis et al.^[22]^	Greece	21.40 ± 2.9/23.2 ± 3.7	28.4 ± 2.7/28.9 ± 2.3	10/20	†/‡	6	# **
Di Rosa et al.^[23]^	Rome	45.08 ± 14.19/45.5 ± 11.63	32.14 ± 4.68/33.54 ± 5.49	133/135	†/§	4–12	# ** ††
Volek et al.^[24]^	USA	33.62 ± 2.62/33.62 ± 2.62	31.68 ± 2.52/31.68 ± 2.52	15/13	§/‡	3	#
Moreno et al.^[25]^	Spain	44.4 ± 8.6/46.3 ± 9.3	35.1 ± 4.5/35.1 ± 5.3	27/26	∥/*	48	# ** ††
Michalczyk et al.^[26]^	Poland	42 ± 7/41 ± 6	32.52 ± 4.50/33.21 ± 4.55	46/45	∥/¶	12	# ** ††
Buga et al.^[27]^	USA	35 ± 3/35 ± 3	31.2 ± 0.7/30.9 ± 0.7	25/12	∥/‡	6	# ††
McManus et al.^[28]^	USA	44 ± 10/44 ± 10	34 ± 5/33 ± 3	31/30	†/‡	72	# ** ††
Barnard et al.^[29]^	USA	57.4/55.6	33.6 ± 5.2/32.6 ± 3.3	29/30	‡/*	14	# ** ††
Luscombe-Marsh et al.^[30]^	Australia	51.5 ± 2.5/49 ± 3	33.3 ± 0.9/34.6 ± 0.9	27/30	‡/¶	16	# **
Petersen et al.^[31]^	European	36 ± 8/37 ± 8	35.5 ± 4.9/35.6 ± 4.9	336/312	‡/¶	10	# ** ††
Tapsell et al.^[32]^	Australia	44.3 ± 10.4/44.2 ± 11.2	31.4 ± 3.8/30.8 ± 3.7	47/48	‡/*	12	#
Shikany et al.^[33]^	USA	40.2 ± 9.2/39.7 ± 9.1	40.4 ± 3.8/41.4 ± 3.8	50/45	‡/*	52	# ** ††
Psota et al.^[34]^	USA	38.8 ± 0.8/39.0 ± 0.9	31.0 ± 0.6/30.6 ± 0.6	32/28	‡/¶	48	# ** ††
Kahleova et al.^[35]^	USA	58.3/56.6	33.7/34.3	30/32	‡/†	36	#
Barnard et al.^[36]^	USA	58.3 ± 8.4/56.6 ± 10.9	33.7 ± 3.4/34.3 ± 2.7	30/32	‡/†	16	# **

BMI = body mass index.

* Low-calorie diet.

† Mediterranean diet.

‡ Low-fat diet.

§ Very low calorie ketogenic diet.

∥ Ketogenic diet.

¶ Moderate-fat diet.

# Body weight.

** Body mass index.

†† Waist circumference.

### 3.3. Risk of bias assessment

**Figure 2.**
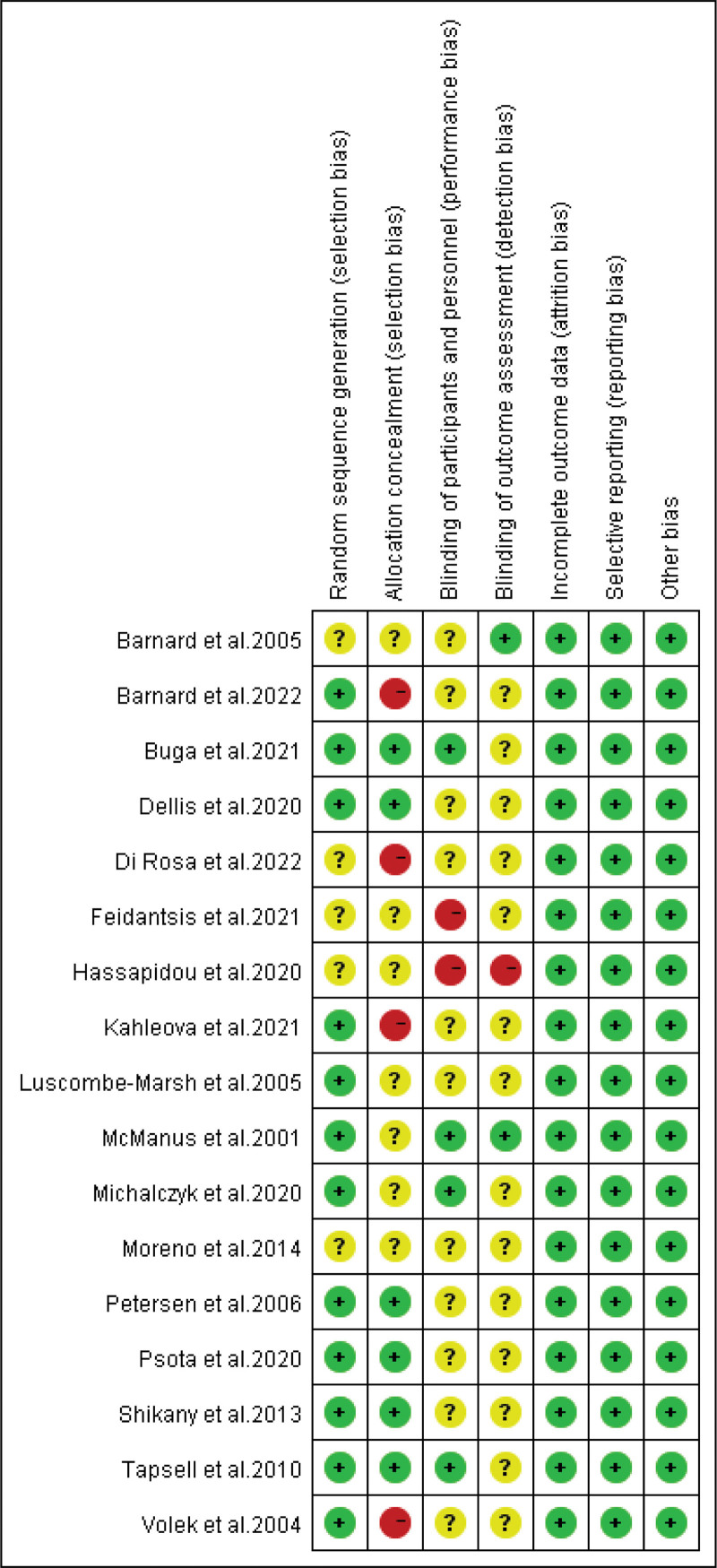
Risk of bias assessment.

Using Review Manager 5.4.1 software, 17 randomized controlled trials were evaluated. 12 studies disclosed random allocation methods, 6 described allocation concealment, and 6 detailed blinding procedures. There were no reported instances of loss to follow-up or compromised data integrity, indicating an absence of other bias (Fig. 2).

### 3.4. Body weight

#### 3.4.1. Network evidence

**Figure 3.**
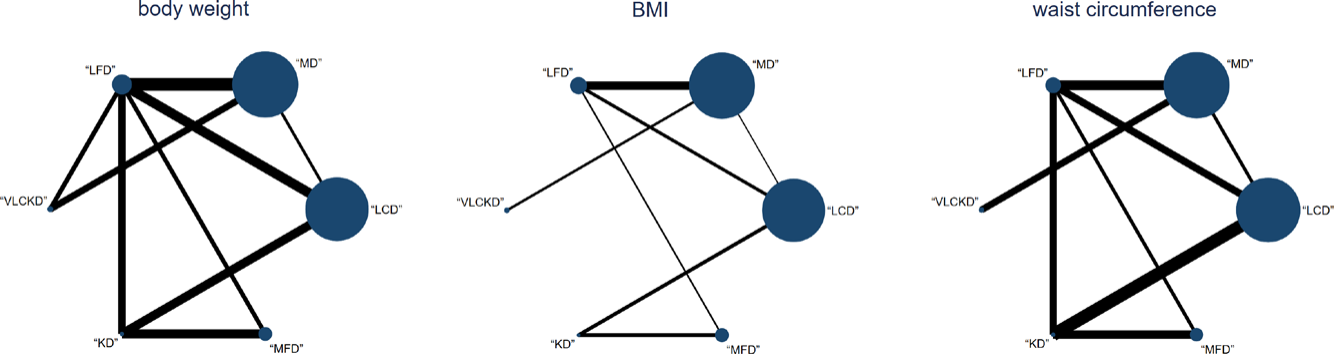
Network evidence diagram. BMI = body mass index, KD = ketogenic diet, LCD = low-calorie diet, LFD = low-fat diet, MD = Mediterranean diet, MFD = moderate-fat diet, VLCKD = very low calorie ketogenic diet.

A total of the network meta-analysis included 17 studies, representing various interventions. The sample size of is denoted by the point size, and the amount of research by the line thickness. A closed loop was formed, necessitating inconsistency checks (Fig. 3). The global inconsistency test showed that *P* = .233, indicating that there was no overall inconsistency (Fig. S1a, Supplemental Digital Content). http://links.lww.com/MD/N594 However, the local inconsistency test indicated significant inconsistency (*P* < .05) between certain treatment methods (Fig. S2a, Supplemental Digital Content). http://links.lww.com/MD/N594

#### 3.4.2. Network meta-analysis and probability ranking

**Figure 4.**
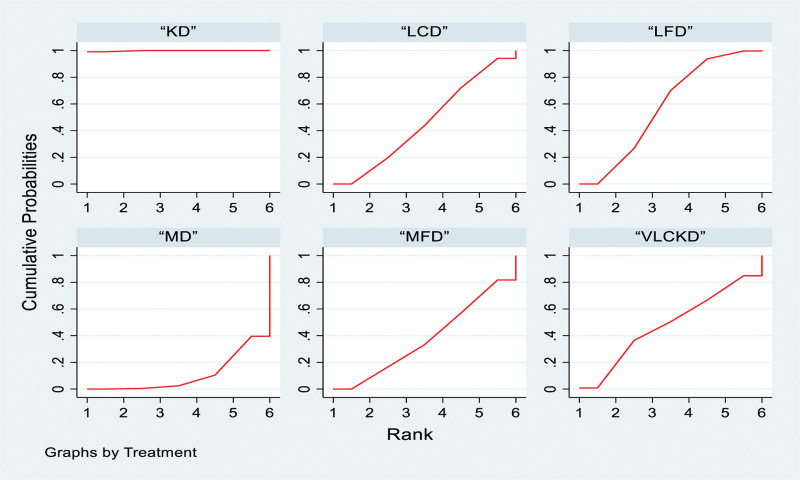
Network meta-analysis ranking results (body weight). KD = ketogenic diet, LCD = low-calorie diet, LFD = low-fat diet, MD = Mediterranean diet, MFD = moderate-fat diet, VLCKD = very low calorie ketogenic diet.

The ketogenic diet outperformed the low-fat diet, very low-calorie ketogenic diet, low-calorie diet, medium-fat diet and Mediterranean diet, with odds ratios (ORs) and 95% confidence intervals (CIs) as follows: 8.33 (3.49, 13.17), 8.64 (1.61, 15.67), 8.87 (3.74, 14.00), 9.35 (4.11, 14.60), and 11.07 (5.68, 16.46), respectively. No significant differences were observed between the other groups (Table 2). For probability ranking, 6 types of interventions were ranked from Rank 1 to 6. The ketogenic diet ranked highest at 99.8%, followed by low-fat diet (58%), very low-calorie ketogenic diet (47.8%), low-calorie diet (46%), medium-fat diet (37.7%) and Mediterranean diet (10.6%; Fig. 4).

**Table 2:** Network meta-analysis results for body weight (lower left corner) and BMI (upper right corner) [OR (95 % CI)].

Ketogenic diet	4.40 (2.44, 6.37)	5.21 (2.16, 8.26)	4.50 (2.56, 6.43)	4.66 (2.71, 6.61)	5.14 (3.04, 7.25)
8.33 (3.49, 13.17)	Low-fat diet	0.81 (−1.66, 3.27)	0.09 (−0.99, 1.18)	0.25 (−0.79, 1.29)	0.74 (−0.37, 1.85)
8.64 (1.61, 15.67)	0.31 (−4.87, 5.50)	Very low calorie ketogenic diet	0.71 (−1.75, 3.18)	0.56 (−2.10, 3.22)	−0.07 (−2.27, 2.13)
8.87 (3.74, 14.00)	0.54 (−2.44, 3.52)	0.23 (−5.52, 5.98)	low-calorie diet	0.16 (−1.28, 1.60)	0.65 (−0.46, 1.75)
9.35 (4.11, 14.60)	1.02 (−2.42, 4.47)	0.71 (−5.50, 6.92)	0.48 (−3.95, 4.92)	Moderate-fat diet	0.49 (−1.00, 1.98)
11.07 (5.68, 16.46)	2.74 (−0.01, 5.49)	2.43 (−2.82, 7.67)	2.20 (−1.07, 5.47)	1.72 (−2.66, 6.09)	Mediterranean diet

### 3.5. Body mass index

#### 3.5.1. Network evidence

The analysis formed a closed loop, necessitating inconsistency testing (Fig. 3). The global inconsistency test showed *P* = .347, indicating no inconsistency (Fig. S1b, Supplemental Digital Content). http://links.lww.com/MD/N594 Local inconsistency test also found no significant inconsistency (*P* > .05; Fig. S2b, Supplemental Digital Content). http://links.lww.com/MD/N594

#### 3.5.2. Network meta-analysis and probability ranking

**Figure 5.**
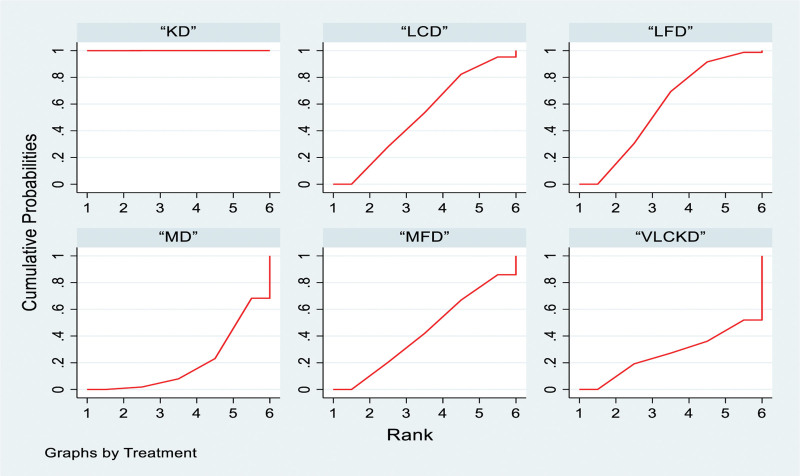
Network meta-analysis ranking results (BMI). BMI = body mass index, KD = ketogenic diet, LCD = low-calorie diet, LFD = low-fat diet, MD = Mediterranean diet, MFD = moderate-fat diet, VLCKD = very low calorie ketogenic diet.

The ketogenic diet demonstrated superiority over the low-fat diet, very low-calorie ketogenic diet, low-calorie diet, medium-fat diet and Mediterranean diet, with ORs (95% CI) of 4.40 (2.44, 6.37), 5.21 (2.16, 8.26), 4.50 (2.56, 6.43), 4.66 (2.71, 6.61) and 5.14 (3.04, 7.25), respectively. There were no significant differences between the other comparisons (Table 2). In the probability ranking, the interventions were ranked in 6 levels. The ketogenic diet achieved the highest ranking at 100%, followed by the low-fat diet (58%), low-calorie diet (51.8%), medium-fat diet (43%), very low-calorie ketogenic diet (26.9%), and Mediterranean diet (20.2%; Fig. 5).

### 3.6. Waist circumference

#### 3.6.1. Network evidence

The formation of a closed loop within the study necessitated inconsistency detection (Fig. 3). The global inconsistency test produced a *P* value of .069, suggesting no overall inconsistency (Fig. S1c, Supplemental Digital Content). http://links.lww.com/MD/N594 However the local inconsistency test revealed significant discrepancies (*P* < .05) between 2 treatment groups, indicating localized inconsistency (Fig. S2c, Supplemental Digital Content). http://links.lww.com/MD/N594

#### 3.6.2. Network meta-analysis and probability ranking

**Figure 6.**
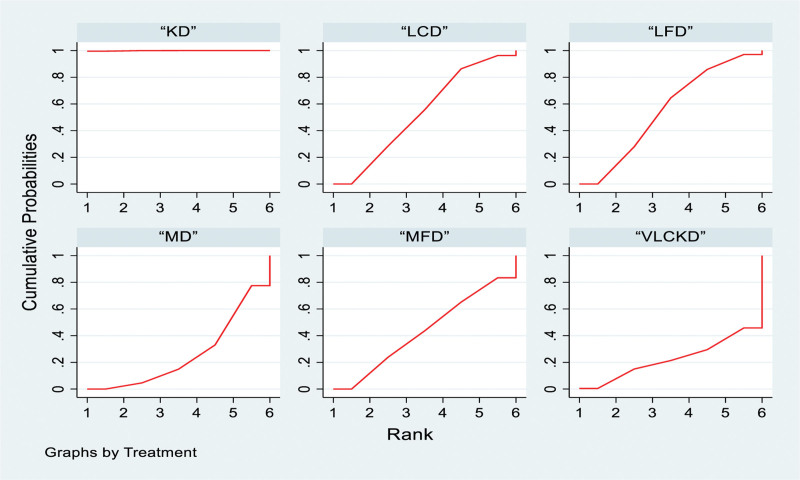
Network meta-analysis ranking results (waist circumference). KD = ketogenic diet, LCD = low-calorie diet, LFD = low-fat diet, MD = Mediterranean diet, MFD = moderate-fat diet, VLCKD = very low calorie ketogenic diet.

In the network meta-analysis the ketogenic diet outperformed the low-fat diet, low-calorie diet, medium-fat diet, Mediterranean diet and very low-calorie ketogenic diet, with ORs and 95% confidence levels (CIs) of 9.02 (4.27, 13.76), 9.15 (3.79, 14.50), 9.76 (4.58, 14.94), 11.18 (4.89, 17.48) and 12.30 (2.92, 21.68), respectively. No significant differences were observed between the other group comparisons (Table 3). The probabilistic ranking, divided into 6 levels, indicating the following order from the most to least effective: ketogenic diet (99.9%), low-fat diet (55.1%), low-calorie diet (53.4%), medium-fat diet (43.2%), Mediterranean diet (26%), and very low-calorie ketogenic diet (22.4%; Fig. 6).

**Table 3:** Network meta-analysis results for waist circumference [OR (95 % CI)].

Ketogenic diet					
9.02 (4.27, 13.76)	Low-fat diet				
9.15 (3.79, 14.50)	0.13 (−3.61, 3.86)	Low-calorie diet			
9.76 (4.58, 14.94)	0.74 (−3.19,4.67)	0.61 (−4.64,5.87)	Moderate-fat diet		
11.18 (4.89, 17.48)	2.16 (−2.71, 7.03)	2.04 (−1.94, 6.01)	1.42 (−4.77, 7.62)	Mediterranean diet	
12.30 (2.92, 21.68)	3.28 (−5.21, 11.77)	3.16 (−4.85, 11.17)	2.54 (−6.77, 11.86)	1.12 (−5.84, 8.08)	Very low calorie ketogenic diet

### 3.7. Publication bias

Publication bias was assessed using funnel plot generated by Stata 16.0 software. For body weight (BW), studies predominantly aligned to the right side of the funnel plot, clustering in the middle and upper regions, suggesting publication bias (Fig. S3a, Supplemental Digital Content). http://links.lww.com/MD/N594 Body mass index (BMI) studies displayed a roughly symmetrical distribution across the funnel plot, primarily concentrated in the middle and upper sections, indicating potential small-sample publication bias (Fig. S3b, Supplemental Digital Content). http://links.lww.com/MD/N594 Waist circumference (WC) studies also showed a roughly symmetrical distribution, with a concentration in the middle and upper parts of the funnel plot, again suggesting small-sample publication bias (Fig. S3c, Supplemental Digital Content). http://links.lww.com/MD/N594

## 4. Discussion

### 4.1. Evidence summary

Network meta-analysis, an evolution of traditional meta-analysis, facilitates the comparison of various factors and interventions for the same condition, enabling quantitative statistical analysis.^[37]^

Obesity has emerged as a significant global public health issue, associated with various chronic diseases including cardiovascular disease,^[38,39]^ diabetes,^[40,41]^ nonalcoholic fatty liver disease (NAFLD),^[42–44]^ various cancers,^[45,46]^ chronic renal failure,^[47]^ and musculoskeletal diseases.^[48]^ The obesity and overweight epidemic not only increases morbidity and mortality rates, but also escalates complication incidences, degrades quality of life, and inflates healthcare costs.^[49,50]^ Maintaining optimal body weight is the basic and best measure to prevent these conditions.^[51,52]^

The network meta-analysis compared different dietary interventions on obesity, analyzing outcomes based on BW, BMI, and WC. The ketogenic diet was found to be the most effective, particularly when compared with the very low-calorie ketogenic and medium-fat diets, which served as indirect comparison bridges. The ketongenic diet consistently ranked highest for the effectiveness in reducing BW, BMI and WC, followed by the low-fat and low-calorie diets, with the Mediterranean diet ranking lowest. This aligns with findings from Choi et al,^[53]^ which demonstrates the superior effectiveness of the ketogenic diet in weight reduction over low-fat diet, though without significant differences in BMI and WC. The ketogenic diet induces a state of metabolic hunger by sererely limiting carbohydrates intake and increasing fat consumption, which, according to Johnston et al,^[54]^ can lead to significant weight loss (an average of 6.34 kilograms) through appetite and hunger regulation. However, the long-term sustainability and effectiveness of the ketogenic diet for weight loss require careful consideration. Landry et al^[55]^ highlighted the importance of adherence to the ketogenic diet, suggesting that its safety and effectiveness must be assured before recommending it widely for obesity treatment. Clinicians should consider these findings when advising dietary intervention for obese patients.

### 4.2. Limitations

This study, while employing network meta-analysis, encountered limitations due to the modest volume and dated nature of the literature, which may have compromised the statistical robustness of the findings. Furthermore, the control measures in the included studies were not consistently stringent. The amalgamation of short-term and long-term studies without differentiation could lead to variability in the results. The overall methodological quality of the studies was moderate, introducing a potential risk of bias. Literature searches were limited to 4 Chinese and 4 English databases, possibly leading to incomplete data collection and an inherent risk of bias. Local inconsistency was detected, and the absence of subgroup analysis may limit the interpretability of the results. Additionally, the geographic diversity of the studies, including countries like Greece, the United States, Spain and Australia, introduced variations in environmental and dietary factors that could influence the outcomes.

## 5. Conclusion

This study systematically evaluated the effectiveness of common dietary interventions (ketogenic diet, low-fat diet, Mediterranean diet and low-calorie diet) for obesity management. Network meta-analysis findings indicated that the ketogenic diet had the highest likelihood of ranking as the most effectiveness intervention in terms of body weight, body mass index, and waist circumference, followed by the low-fat diet, low-calorie diet, and Mediterranean diet. However, due to the limited quantity and quality of the included studies, these conclusions need to be further verified by more high-quality randomized controlled trials.

## Author contributions

**Data curation:** Tianrong Liao, Jiayu Su.

**Formal analysis:** Tianrong Liao, Jiayu Su, Tingwei Quan.

**Investigation:** Jiayu Su, Tingwei Quan, Yu Luo, Yiqian Zeng.

**Resources:** Tianrong Liao, Jiayu Su.

**Visualization:** Tianrong Liao, Tingwei Quan.

**Writing – original draft:** Tianrong Liao.

**Writing – review & editing:** Dandan Chen, Hongzhen Tang.

## Supplementary Material


