# Supplementary material for: The impact of 3 different dietary interventions on overweight or obese adults: A network meta-analysis
Source: Medicine (Baltimore). 2024 Oct 18;103(42):e39749. doi: 10.1097/MD.0000000000039749 (PMC11495794; doi:10.1097/MD.0000000000039749)
Supplement: Supplementary file 1 [file medi-103-e39749-s001.pdf]

Supplemental File.1 Search strategy

PubMed search strategy

#1. "Obesity"[Mesh] 267214

#2. (((((((((((((Overweight[Title/Abstract]) OR (Over fat[Title/Abstract])) OR (Body Weight[Title/Abstract])) OR (Body Weights[Title/Abstract])) OR (Weight, Body[Title/Abstract])) OR (Weights, Body[Title/Abstract])) OR (weight loss[Title/Abstract])) OR (Loss, Weight[Title/Abstract])) OR (Losses, Weight[Title/Abstract])) OR (Weight Losses[Title/Abstract])) OR (Weight Reduction[Title/Abstract])) OR (Reduction, Weight[Title/Abstract])) OR (Reductions, Weight[Title/Abstract])) OR (Weight Reductions[Title/Abstract])) OR (Bariatrics[Title/Abstract])
427609

#3. ("Obesity"[Mesh]) OR ((((((((((((((Overweight[Title/Abstract]) OR (Over fat[Title/Abstract])) OR (Body Weight[Title/Abstract])) OR (Body Weights[Title/Abstract])) OR (Weight, Body[Title/Abstract])) OR (Weights, Body[Title/Abstract])) OR (weight loss[Title/Abstract])) OR (Loss, Weight[Title/Abstract])) OR (Losses, Weight[Title/Abstract])) OR (Weight Losses[Title/Abstract])) OR (Weight Reduction[Title/Abstract])) OR (Reduction, Weight[Title/Abstract])) OR (Reductions, Weight[Title/Abstract])) OR (Weight Reductions[Title/Abstract])) OR (Bariatrics[Title/Abstract]))
602424

#4. "Adult"[Mesh] 8008487

#5. Adults[Title/Abstract] 777959

#6. ("Adult"[Mesh]) OR (Adults[Title/Abstract]) 8266779

#7. "Diet, Mediterranean"[Mesh] 5480

#8. ((Mediterranean Diet[Title/Abstract]) OR (Diets, Mediterranean[Title/Abstract])) OR (Mediterranean Diets[Title/Abstract]) 8114

#9. ("Diet, Mediterranean"[Mesh]) OR (((Mediterranean Diet[Title/Abstract]) OR (Diets, Mediterranean[Title/Abstract])) OR (Mediterranean Diets[Title/Abstract])) 9121

#10. "Diet, Ketogenic"[Mesh] 2436

#11. ((Ketogenic Diet[Title/Abstract]) OR (Diets, Ketogenic[Title/Abstract])) OR (Ketogenic Diets[Title/Abstract]) 4710

#12. ("Diet, Ketogenic"[Mesh]) OR (((Ketogenic Diet[Title/Abstract]) OR (Diets, Ketogenic[Title/Abstract])) OR (Ketogenic Diets[Title/Abstract])) 4953

#13. "Diet, Fat-Restricted"[Mesh] 4052

#14. (((((((((((((Diet, Fat Restricted[Title/Abstract]) OR (Fat-Restricted Diet[Title/Abstract])) OR (Diets, Fat-Restricted[Title/Abstract])) OR (Fat Restricted Diet[Title/Abstract])) OR (Fat-Restricted Diets[Title/Abstract])) OR (Diet, Low-Fat[Title/Abstract])) OR (Diet, Low Fat[Title/Abstract])) OR (Diets, Low-Fat[Title/Abstract])) OR (Low-Fat Diet[Title/Abstract])) OR (Low-Fat Diets[Title/Abstract])) OR (Diet, Fat-Free[Title/Abstract])) OR (Diet, Fat Free[Title/Abstract])) OR (Diets, Fat-Free[Title/Abstract])) OR (Fat-Free Diet[Title/Abstract])) OR (Fat-Free Diets[Title/Abstract])
8693

#15. ("Diet, Fat-Restricted"[Mesh]) OR (((((((((((((((Diet, Fat Restricted[Title/Abstract]) OR (Fat-Restricted Diet[Title/Abstract])) OR (Diets, Fat-Restricted[Title/Abstract])) OR (Fat Restricted Diet[Title/Abstract])) OR (Fat-Restricted Diets[Title/Abstract])) OR (Diet, Low-Fat[Title/Abstract])) OR (Diet, Low Fat[Title/Abstract])) OR (Diets, Low-Fat[Title/Abstract])) OR (Low-Fat Diet[Title/Abstract])) OR (Low-Fat Diets[Title/Abstract])) OR (Diet, Fat-Free[Title/Abstract])) OR (Diet, Fat Free[Title/Abstract])) OR (Diets, Fat-Free[Title/Abstract])) OR (Fat-Free Diet[Title/Abstract])) OR (Fat-Free Diets[Title/Abstract]))
11331

#16. ((("Diet, Mediterranean"[Mesh]) OR (((Mediterranean Diet[Title/Abstract]) OR (Diets, Mediterranean[Title/Abstract])) OR (Mediterranean Diets[Title/Abstract]))) OR (("Diet, Ketogenic"[Mesh]) OR (((Ketogenic Diet[Title/Abstract]) OR (Diets, Ketogenic[Title/Abstract])) OR (Ketogenic Diets[Title/Abstract])))) OR (("Diet, Fat-Restricted"[Mesh]) OR (((((((((((((((Diet, Fat Restricted[Title/Abstract]) OR (Fat-Restricted Diet[Title/Abstract])) OR (Diets, Fat-Restricted[Title/Abstract])) OR (Fat Restricted Diet[Title/Abstract])) OR (Fat-Restricted Diets[Title/Abstract])) OR (Diet, Low-Fat[Title/Abstract])) OR (Diet, Low Fat[Title/Abstract])) OR (Diets, Low-Fat[Title/Abstract])) OR (Low-Fat Diet[Title/Abstract])) OR (Low-Fat Diets[Title/Abstract])) OR (Diet, Fat-Free[Title/Abstract])) OR (Diet, Fat Free[Title/Abstract])) OR (Diets, Fat-Free[Title/Abstract])) OR (Fat-Free Diet[Title/Abstract])) OR (Fat-Free Diets[Title/Abstract])))
24775

#17. randomized controlled trial[Publication Type] OR randomized[Title/Abstract] OR placebo[Title/Abstract] 1045061

#18. (((("Obesity"[Mesh]) OR (((((((((((((((Overweight[Title/Abstract]) OR (Over fat[Title/Abstract])) OR (Body Weight[Title/Abstract])) OR (Body Weights[Title/Abstract])) OR (Weight, Body[Title/Abstract])) OR (Weights, Body[Title/Abstract])) OR (weight loss[Title/Abstract])) OR (Loss, Weight[Title/Abstract])) OR (Losses, Weight[Title/Abstract])) OR (Weight Losses[Title/Abstract])) OR (Weight Reduction[Title/Abstract])) OR (Reduction, Weight[Title/Abstract])) OR (Reductions, Weight[Title/Abstract])) OR (Weight Reductions[Title/Abstract])) OR (Bariatrics[Title/Abstract]))) AND (("Adult"[Mesh]) OR (Adults[Title/Abstract]))) AND (((("Diet, Mediterranean"[Mesh]) OR (((Mediterranean Diet[Title/Abstract]) OR (Diets, Mediterranean[Title/Abstract])) OR (Mediterranean Diets[Title/Abstract]))) OR (("Diet, Ketogenic"[Mesh]) OR (((Ketogenic Diet[Title/Abstract]) OR (Diets, Ketogenic[Title/Abstract])) OR (Ketogenic Diets[Title/Abstract])))) OR (("Diet, Fat-Restricted"[Mesh]) OR (((((((((((((((Diet, Fat Restricted[Title/Abstract]) OR (Fat-Restricted Diet[Title/Abstract])) OR (Diets, Fat-Restricted[Title/Abstract])) OR (Fat Restricted Diet[Title/Abstract])) OR (Fat-Restricted Diets[Title/Abstract])) OR (Diet, Low-Fat[Title/Abstract])) OR (Diet, Low Fat[Title/Abstract])) OR (Diets, Low-Fat[Title/Abstract])) OR (Low-Fat Diet[Title/Abstract])) OR (Low-Fat Diets[Title/Abstract])) OR (Diet, Fat-Free[Title/Abstract])) OR (Diet, Fat Free[Title/Abstract])) OR (Diets, Fat-Free[Title/Abstract])) OR (Fat-Free Diet[Title/Abstract])) OR (Fat-Free Diets[Title/Abstract]))))) AND (randomized controlled trial[Publication Type] OR randomized[Title/Abstract] OR placebo[Title/Abstract]) 1158

Web of Science search strategy

#1. TS=(Obesity OR Overweight OR Over fat OR Body Weight OR Body Weights OR Weight, Body OR Weights, Body OR weight loss OR Loss, Weight OR Losses, Weight OR Weight Losses OR Weight Reduction OR Reduction, Weight OR Reductions, Weight OR Weight Reductions OR Bariatrics) 1120890

#2. TS=(Adult OR Adults) 1984827

#3. TS=(Diet, Mediterranean OR Mediterranean Diet OR Diets, Mediterranean OR Mediterranean Diets OR Diet, Ketogenic OR Ketogenic Diet OR Diets, Ketogenic OR Ketogenic Diets OR Diet, Fat-Restricted OR Diet, Fat Restricted OR Fat-Restricted Diet OR Diets, Fat-Restricted OR Fat Restricted Diet OR Fat-Restricted Diets OR Diet, Low-Fat OR Diet, Low Fat OR Diets, Low-Fat OR Low-Fat Diet OR Low-Fat Diets OR Diet, Fat-Free OR Diet, Fat Free OR Diets, Fat-Free OR Fat-Free Diet OR Fat-Free Diets) 88320

#4. TS=(randomized controlled trial OR Randomized OR Placebo OR RCT) 1214334

#5. #1 AND #2 AND #3 AND #4 1639

Embase search strategy

#1. 'obesity'/exp OR obesity 822,923

#2.'overweight':ab,ti OR 'over fat':ab,ti OR 'body weight':ab,ti OR 'body weights':ab,ti OR 'weight, body':ab,ti OR 'weights, body':ab,ti OR 'weight loss':ab,ti OR 'loss, weight':ab,ti OR 'losses, weight':ab,ti OR 'weight losses':ab,ti OR 'weight reduction':ab,ti OR 'reduction, weight':ab,ti OR 'reductions, weight':ab,ti OR 'weight reductions':ab,ti OR 'bariatrics':ab,ti 615,285

#3. #1 OR #2 1,210,317

#4. adult 10,388,755

#5. 'adults':ab,ti 1,031,194

#6. #4 OR #5 10,675,862

#7. diet, AND mediterranean 18,252

#8. 'mediterranean diet':ab,ti OR 'diets, mediterranean':ab,ti OR 'mediterranean diets':ab,ti 10,280

#9. #7 OR #8 18,269

#10. diet, AND ketogenic 9,943

#11. 'ketogenic diet':ab,ti OR 'diets, ketogenic':ab,ti OR 'ketogenic diets':ab,ti 6,558

#12. #10 OR #11 9,989

#13. diet, AND 'fat restricted' 207

#14. 'diet, fat restricted':ab,ti OR 'fat-restricted diet':ab,ti OR 'diets, fat-restricted':ab,ti OR 'fat restricted diet':ab,ti OR 'fat-restricted diets':ab,ti OR 'diet, low-fat':ab,ti OR 'diet, low fat':ab,ti OR 'diets, low-fat':ab,ti OR 'low-fat diet':ab,ti OR 'low-fat diets':ab,ti OR 'diet, fat-free':ab,ti OR 'diet, fat free':ab,ti OR 'diets, fat-free':ab,ti OR 'fat-free diet':ab,ti OR 'fat-free diets':ab,ti

8,166
#15. #13 OR #14 8,222
#16. #9 OR #12 OR #15 35,595
#17. 'randomized controlled trial':ab,ti OR 'randomized':ab,ti OR 'placebo':ab,ti OR 'rct':ab,ti 1,190,283
#18. #3 AND #6 AND #16 AND #17 963

Cochrane Library search strategy

#1 Obesity 52391
#2 (Overweight):ab,ti,kw OR (Over fat):ab,ti,kw OR (Body Weight):ab,ti,kw OR (Body Weights):ab,ti,kw OR (Weight, Body):ab,ti,kw OR (Weights, Body):ab,ti,kw OR (weight loss):ab,ti,kw OR (Loss, Weight):ab,ti,kw OR (Losses, Weight):ab,ti,kw OR (Weight Losses):ab,ti,kw OR (Weight Reduction):ab,ti,kw OR (Reduction, Weight):ab,ti,kw OR (Reductions, Weight):ab,ti,kw OR (Weight Reductions):ab,ti,kw OR (Bariatrics):ab,ti,kw 115158
#3 #1 OR #2 134922
#4 Adult 780420
#5 (Adults):ab,ti,kw 135326
#6 #4 OR #5 841205
#7 Diet, Mediterranean 2697
#8 (Mediterranean Diet):ab,ti,kw OR (Diets, Mediterranean):ab,ti,kw OR (Mediterranean Diets):ab,ti,kw 2590
#9 #7 OR #8 2710
#10 Diet, Ketogenic 786
#11 (Ketogenic Diet):ab,ti,kw OR (Diets, Ketogenic):ab,ti,kw OR (Ketogenic Diets):ab,ti,kw 757
#12 #10 OR #11 797
#13 Diet, Fat-Restricted 1195
#14 (Diet, Fat Restricted):ab,ti,kw OR (Fat-Restricted Diet):ab,ti,kw OR (Diets, Fat-Restricted):ab,ti,kw OR (Fat Restricted Diet):ab,ti,kw OR (Fat-Restricted Diets):ab,ti,kw OR (Diet, Low-Fat):ab,ti,kw OR (Diet, Low Fat):ab,ti,kw OR (Diets, Low-Fat):ab,ti,kw OR (Low-Fat Diet):ab,ti,kw OR (Low-Fat Diets):ab,ti,kw OR (Diet, Fat-Free):ab,ti,kw OR (Diet, Fat Free):ab,ti,kw OR (Diets, Fat-Free):ab,ti,kw OR (Fat-Free Diet):ab,ti,kw OR (Fat-Free Diets):ab,ti,kw 10288
#15 #13 OR #14 10301
#16 #9 OR #12 OR #15 12843
#17 (randomized controlled trial):ab,ti,kw OR (Randomized):ab,ti,kw OR (Placebo):ab,ti,kw OR (RCT):ab,ti,kw 1256899
#18 #3 AND #6 AND #16 AND #17 4235

CBM search strategy

#1. "肥胖症"[不加权:扩展] 292085
#2. "肥胖"[常用字段:智能] OR "超重"[常用字段:智能] OR "减肥"[常用字段:智能] OR "减重"[常用

字段:智能] OR "减脂"[常用字段:智能] OR "降脂"[常用字段:智能] OR "体重"[常用字段:智能] 971418

#3. ("肥胖"[常用字段:智能] OR "超重"[常用字段:智能] OR "减肥"[常用字段:智能] OR "减重"[常用字段:智能] OR "减脂"[常用字段:智能] OR "降脂"[常用字段:智能] OR "体重"[常用字段:智能]) OR ("肥胖症"[不加权:扩展])974083

#4. "地中海饮食"[常用字段:智能] OR "生酮饮食"[常用字段:智能] OR "低脂饮食"[常用字段:智能] 1927

#5. "随机对照试验"[不加权:扩展] 201330

#6. "随机对照实验"[常用字段:智能] OR "随机对照研究"[常用字段:智能] OR "RCT"[常用字段:智能] OR "随机对照"[常用字段:智能] OR "随机"[常用字段:智能] 2225552

#7. ("随机对照实验"[常用字段:智能] OR "随机对照研究"[常用字段:智能] OR "RCT"[常用字段:智能] OR "随机对照"[常用字段:智能] OR "随机"[常用字段:智能]) OR ("随机对照试验"[不加权:扩展]) 2225767

#8. (("随机对照实验"[常用字段:智能] OR "随机对照研究"[常用字段:智能] OR "RCT"[常用字段:智能] OR "随机对照"[常用字段:智能] OR "随机"[常用字段:智能]) OR ("随机对照试验"[不加权:扩展])) AND ("地中海饮食"[常用字段:智能] OR "生酮饮食"[常用字段:智能] OR "低脂饮食"[常用字段:智能]) AND (("肥胖"[常用字段:智能] OR "超重"[常用字段:智能] OR "减肥"[常用字段:智能] OR "减重"[常用字段:智能] OR "减脂"[常用字段:智能] OR "降脂"[常用字段:智能] OR "体重"[常用字段:智能]) OR ("肥胖症"[不加权:扩展])) 140

VIP search strategy

#1. （中英文扩展）题名或关键词=肥胖 OR 超重 OR 减肥 OR 减重 OR 减脂 OR 降脂 OR 体重

AND

（中英文扩展）文摘=地中海饮食 OR 生酮饮食 OR 低脂饮食

AND

（中英文扩展）文摘=随机对照试验 OR 随机对照实验 OR 随机对照研究 OR RCT OR 随机对照 OR 随机 66

CNKI search strategy

#1. （中英文扩展）（主题：肥胖）OR（主题：超重）OR（主题：减肥）OR（主题：减重）OR（主题：减脂）OR（主题：降脂）OR（主题：体重）

AND

（中英文扩展）（篇关摘：地中海饮食(精确)）OR（篇关摘：生酮饮食(精确)）OR（篇关摘：低脂饮食(精确)）

AND

（中英文扩展）（篇关摘：随机对照试验(精确)）OR（篇关摘：随机对照实验(精确)）OR（篇关摘：随机对照研究(精确)）OR（篇关摘：RCT(精确)）OR（篇关摘：随机对照(精确)）OR（篇关摘：随机(精确)） 188

Wan-fang Data search strategy

#1. （中英文扩展）：

主题:(肥胖 or 超重 or 减肥 or 减重 or 减脂 or 降脂 or 体重)

AND

题名或关键词:(地中海饮食 or 生酮饮食 or 低脂饮食)

AND

主题:(随机对照试验 or 随机对照实验 or 随机对照研究 or RCT or 随机对照 or 随机）

152

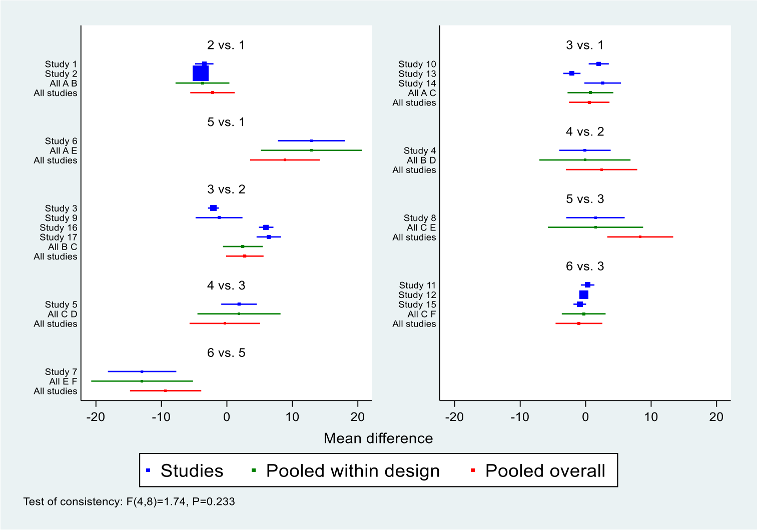

Supplementary Figure 1-a Global inconsistency test results (Body Weight).

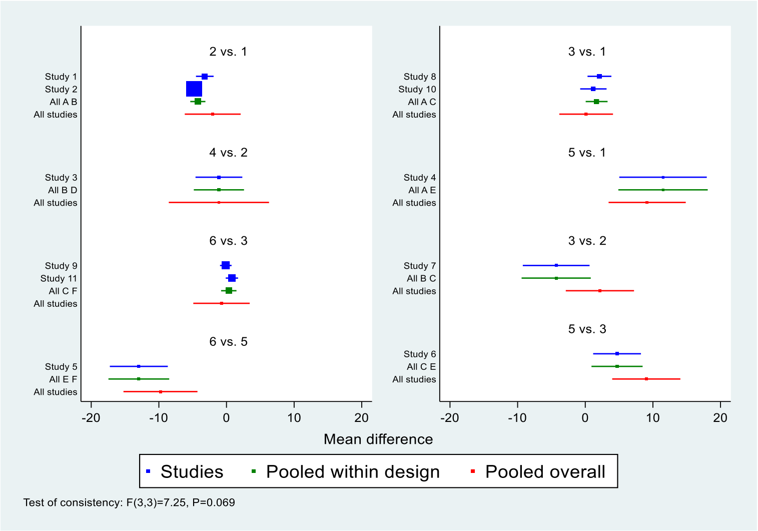

Supplementary Figure 1-c Global inconsistency test results (Waist Circumference).

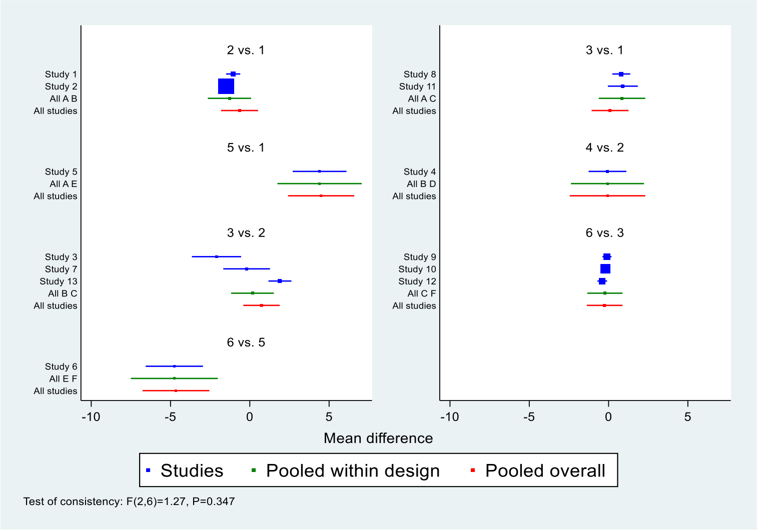

Supplementary Figure 1-b Global inconsistency test results (BMI).

Side	Direct		Indirect		Difference			tau
	Coef.	Std. Err.	Coef.	Std. Err.	Coef.	Std. Err.	P>|z|	
A B	-3.731208	2.335397	-.4936085	2.459309	-3.2376	3.391325	0.340	3.26326
A C	.7551492	2.034282	.2496493	2.568478	.5054998	3.274441	0.877	3.379001
A E	12.9	4.069393	6.178874	3.313548	6.721126	5.247815	0.200	3.122574
B C	2.411493	1.765372	3.46545	2.661947	-1.053956	3.19395	0.741	3.374521
B D	-.1099785	3.792715	4.967565	3.797452	-5.077543	5.366839	0.344	3.219726
C D	1.849823	3.504621	-3.225084	4.064613	5.074907	5.366752	0.344	3.21976
C E	1.501448	3.530109	12.80369	2.863064	-11.30224	4.545017	0.013	2.699489
C F	-.3007881	1.845656	-6.406382	5.056603	6.105594	5.382883	0.257	3.165632
E F	-12.94633	4.137786	-6.844551	3.450834	-6.101775	5.388002	0.257	3.165723

(a)

Side	Direct		Indirect		Difference			tau
	Coef.	Std. Err.	Coef.	Std. Err.	Coef.	Std. Err.	P>|z|	
A B	-1.282928	.5923448	.5313019	.8657403	-1.81423	1.04798	0.083	.8216016
A C	.8453227	.6294668	-.9000981	.7559245	1.745421	.9840909	0.076	.8010366
A E	4.4	1.341902	4.626264	1.62431	-.2262638	2.106913	0.914	1.025287
B C	.2020579	.626872	2.016399	.8469487	-1.814341	1.04809	0.083	.8216283
B D *	-.07	1.124146	1.220045	199.9953	-1.290045	199.9966	0.995	.9460223
C F	-.2330573	.5969961	-.4613335	2.020297	.2282762	2.106662	0.914	1.025263
E F	-4.759808	1.378489	-4.53252	1.593491	-.2272884	2.107009	0.914	1.025289

* Warning: all the evidence about these contrasts comes from the trials which directly compare t
> hem.

(b)

Side	Direct		Indirect		Difference			tau
	Coef.	Std. Err.	Coef.	Std. Err.	Coef.	Std. Err.	P>|z|	
A B	-4.091828	.9668773	6.081497	3.020033	-10.17332	3.168271	0.001	1.273177
A C	1.655211	1.974101	-3.496356	3.17243	5.151566	3.73714	0.168	2.62583
A E	11.5	4.673547	7.736701	3.643903	3.763299	5.926219	0.525	3.308246
B C	-4.299683	2.822604	5.883532	1.444382	-10.18322	3.170687	0.001	1.273503
B D *	-1.12	3.550205	2.953741	200.1145	-4.073741	200.1304	0.984	3.079855
C E	4.7006	2.953223	12.61442	2.784317	-7.91382	4.0587	0.051	2.338815
C F	.3491962	1.952391	-5.936367	4.423978	6.285563	4.835543	0.194	2.723946
E F	-12.95746	3.493822	-6.677031	3.347809	-6.280424	4.838895	0.194	2.72447

* Warning: all the evidence about these contrasts comes from the trials which directly compare t
> hem.

(c)

Supplementary Figure. 2 Local inconsistency test results. (a)Results of body weight; (b)Results of BMI; (c)Results of waist circumference. Annotation: A, Low-calorie diet; B, Mediterranean diet; C, Low-fat diet; D, Very low calorie ketogenic diet; E, Ketogenic diet; F, Moderate-fat diet.

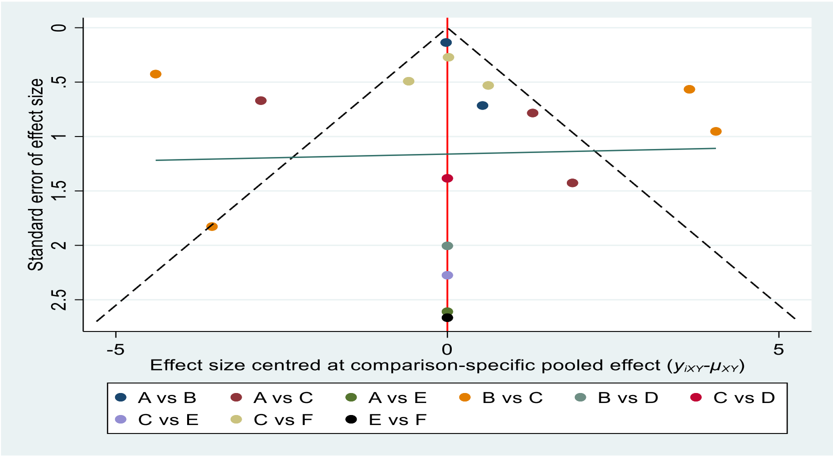

Supplementary Figure 3-a Publication bias funnel plot (Body Weight). Annotation: A, Low-calorie diet; B, Mediterranean diet; C, Low-fat diet; D, Very low calorie ketogenic diet; E, Ketogenic diet; F, Moderate-fat diet.

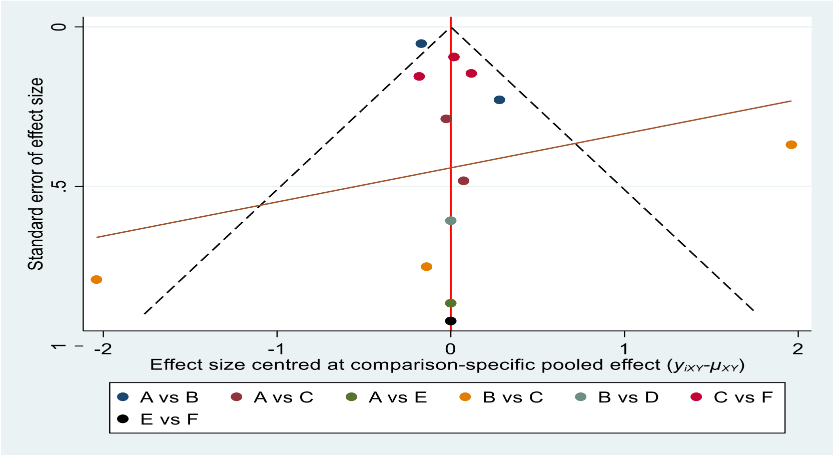

Supplementary Figure 3-b Publication bias funnel plot (BMI). Annotation: A, Low-calorie diet; B, Mediterranean diet; C, Low-fat diet; D, Very low calorie ketogenic diet; E, Ketogenic diet; F, Moderate-fat diet.

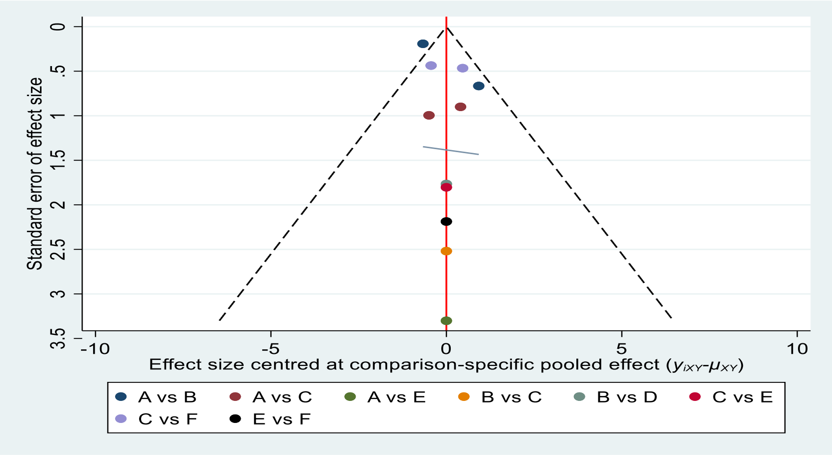

Supplementary Figure 3-c Publication bias funnel plot (Waist Circumference). Annotation: A, Low-calorie diet; B, Mediterranean diet; C, Low-fat diet; D, Very low calorie ketogenic diet; E, Ketogenic diet; F, Moderate-fat diet.
